# Minnesota State Medical Society

**Published:** 1877-08

**Authors:** 


### Reports of Societies.

### MINNESOTA STATE MEDICAL SOCIETY.

The ninth annual meeting of the Minnesota State Medical Society was held in the city of St. Paul on the 19th of June last, continuing for two days, Dr. F. H. Milligan, of Wabasha, presiding.

The meeting was characterized by the usual harmony and good feeling, and, in addition, by the reading of a number of papers and reports of decided ability, as well as by the action of the Society upon various matters, of interest both to our own State and to the profession at large.

The President, Dr. Milligan, in his opening remarks, made certain suggestions of great practical importance; of greater importance, perhaps, to those practiced upon, than to those who practice; although by no means destitute of benefit to the latter class. Of these, the most valuable were recommendations to the Society to adopt measures looking to the enactment of laws by the Legislature making vaccination compulsory, and also, to require all aspirants to pharmaceutical honors to appear before a board of properly qualified examiners for examination and license, with a view to the adoption of a higher standard of education in this important branch.

The President also urged the Society to request our congressional delegation to use every proper means to secure an appropriation from the United States for the families of the discoverers of anæsthetics, as a suitable though tardy recognition of the inestimable benefits conferred by them upon mankind.

These suggestions were unanimously adopted, and committees were appointed to carry their provisions into effect.

Unanimous action was also taken by the Society upon certain recommendations of the Executive Committee, in accordance with which one committee was appointed to coöperate with other organizations in seeking to abolish the duty on quinia, and a second, to endeavor to obtain reduced fares from the railroads in this State for delegates to the meeting of the American Medical Association at Buffalo next year. From all which it will appear that the physicians of this State are in full accord with their brethren in their efforts to promote the interests of humanity, and of the medical profession.

The Society took occasion to reaffirm their previously recorded opinion, that illuminating oils of less than 150° F. flash test are unsafe, and to request the Legislature to restore the test to that standard; they having, most unwisely, seen fit to substitute 130° F. last winter for the higher and safer test.

Upon the earnest recommendation of the Secretary of the

State Board of Health, Dr. C. N. Hewitt, of Red Wing, a committee was appointed whose duty will be to collect and collate facts with reference to the use and abuse of alcohol in this State, taking note of age, sex, nationality, heredity, etc., and to report at the next meeting, so that, if possible, a remedy may be discovered for an evil of vast dimensions, and an era of practical reformation be begun.

Among the papers of special interest was one by Dr. I. E. Finch, of Hastings, chairman of the Committee on Practical Medicine, on Myalgia. The doctor treated his subject with great skill, and succeeded in investing what some had been disposed to regard as trite, if not trifling, with a high degree of interest. His argument, after a close pathological study, was conclusive as to the differentiation of myalgia and true rheumatic affections.

Dr. Franklin Staples, of Winona, read a paper upon carcinomatous affections, citing a number of cases, and supplementing his remarks by the exhibition of photographs, crayon sketches, and pathological specimens.

The report of the Committee on Obstetrics,—Dr. W. L. Lincoln, Wabasha, chairman, elicited a lively discussion as to whether death of the child ever results from strangulation by the cord; the debate arising from a case which was reported, of death in utero from a tight knot upon the cord, there being also a loop of cord around the neck. Some difference of opinion existed, but the general sense of the Society was against the fatal issue of the condition in question. Much interest was shown in the relation of a fatal case of flooding, and the alleged agency of chloroform in conducing to the unfortunate result, by favoring relaxation. Upon this point, it seemed to be the opinion of the majority, that cases, in which chloroform is directly chargeable with the death of the patient, are exceedingly rare, but that due caution should always be exercised in its use.

Other papers of merit were read, and other interesting cases reported, of which want of time and space forbids the mention here, but which will appear in detail in the forthcoming volume of Transactions.

As to personal matters, Dr. W. I. Sloan, U. S. A., now on duty in this city as medical director at the headquarters of the department of Dakota, and one of the oldest and most esteemed members of the medical corps of the army, was elected an honorary member of the Society. The pleasantest feature of the session was the adoption of a hearty vote of thanks to the retiring Secretary, Dr. C. E. Smith, of St. Paul, who had served in that onerous capacity for six years with untiring zeal and marked ability, and, as a more substantial recognition of his services, the presentation to him of a copy of Holmes' System of Surgery—a well deserved testimonial.

The officers elected for the coming yeare are:

President, Otis Ayer, M. D., Le Sueur; 1st Vice-President, C. P. Adams, M. D., Hastings; 2d Vice-President, A. C. Wedge, M. D., Albert Lea; 3d Vice-President, E. E. Collins, M. D., Minneapolis; Treasurer, S. B. Sheardown, M. D., Winona; Recording Secretary, C. H. Boardman, M. D., St. Paul; Corresponding Secretary, E. Phillips, M. D., Minneapolis.

